# A database of human predictive patch test data for skin sensitization

**DOI:** 10.1007/s00204-023-03530-3

**Published:** 2023-08-24

**Authors:** Judy Strickland, Jaleh Abedini, David G. Allen, John Gordon, Victoria Hull, Nicole C. Kleinstreuer, Hon-Sum Ko, Joanna Matheson, Hermann-Josef Thierse, James Truax, Jens T. Vanselow, Matthias Herzler

**Affiliations:** 1 Inotiv, Inc, Morrisville, NC USA; 2 United States Consumer Product Safety Commission, Bethesda, MD USA; 3 National Institutes of Health, National Institute of Environmental Health Sciences, Division of Translational Toxicology, National Toxicology Program Interagency Center for the Evaluation of Alternative Toxicological Methods, Research Triangle Park, NC USA; 4 United States Food and Drug Administration, Silver Spring, MD USA; 5 German Federal Institute for Risk Assessment (BfR), Berlin, Germany

**Keywords:** Allergic contact dermatitis; human predictive patch test data, Skin sensitization, Reference data

## Abstract

Critical to the evaluation of non-animal tests are reference data with which to assess their relevance. Animal data are typically used because they are generally standardized and available. However, when regulatory agencies aim to protect human health, human reference data provide the benefit of not having to account for possible interspecies variability. To support the evaluation of non-animal approaches for skin sensitization assessment, we collected data from 2277 human predictive patch tests (HPPTs), i.e., human repeat insult patch tests and human maximization tests, for skin sensitization from 1555 publications. We recorded protocol elements and positive or negative outcomes, developed a scoring system to evaluate each test for reliability, and calculated traditional and non-traditional dose metrics. We also traced each test result back to its original report to remove duplicates. The resulting database, which contains information for 1366 unique substances, was characterized for physicochemical properties, chemical structure categories, and protein binding mechanisms. This database is publicly available on the National Toxicology Program Interagency Center for the Evaluation of Alternative Toxicological Methods website and in the Integrated Chemical Environment to serve as a resource for additional evaluation of alternative methods and development of new approach methodologies for skin sensitization assessments.

**Supplementary Information:**

The online version contains supplementary material available at 10.1007/s00204-023-03530-3.

## Introduction

Allergic contact dermatitis is a delayed hypersensitivity reaction resulting in a painful inflammatory or itchy skin rash after contact with substances against which a sensitized individual has developed a contact allergy. Allergic contact dermatitis has long-term consequences and can decrease quality of life (Bennike et al. 2019; Kalbousi et al. 2019; Olusegun and Martincigh 2021).

Chemicals to which workers and consumers may be exposed should be tested for skin sensitization potential. Based on data from these tests, chemical sponsors and regulatory authorities determine how a substance should be classified for its skin sensitization potential. These evaluations determine the specific hazard labels and hazard information that should be provided on chemical safety data sheets, the specific personal protective equipment necessary when handling the substance, and safe levels of sensitizing substances in products. The tests traditionally used to make these determinations are animal tests: the murine local lymph node assay (OECD 2010) and the guinea pig maximization and Buehler tests (OECD 1992). However, determinations of skin sensitization potential may also be made using data from human predictive patch tests (HPPTs) (Botham et al. 1991). Such tests include the human maximization test (HMT) and the human repeat insult patch test (HRIPT). Both tests involve repeatedly exposing individuals not known to be allergic to a potential skin sensitizer over several weeks. This induction phase of the test is followed by a rest period and then a challenge phase in which the chemical is reapplied to determine whether an allergic response is elicited. The major difference between the two tests is that the HMT tests a smaller number of subjects and incorporates pretreatment with an irritant to enhance skin penetration when testing nonirritating substances. HPPTs are different from diagnostic patch tests that are performed on patients to identify the chemical source of allergic contact dermatitis but do not provide information on the extent and frequency of previous exposure.

Scientific and ethical concerns have motivated the development and evaluation of non-animal and non-human tests for the skin sensitization potential of chemicals. Critical to the evaluation of non-animal tests are reference data from accepted tests such as those described above to serve as a benchmark for assessing the performance of alternative test methods (OECD 2005) and create regulatory trust in these new methods. Animal data are typically used as reference data because they are generally standardized and available. However, when regulatory agencies aim to protect human health, human data provide the benefit of not having to account for possible interspecies variability. Although HPPT data have been used for hazard characterization and risk assessment for decades, their usefulness and limitations have not been analyzed comprehensively or discussed in detail.

A guideline describing the application of defined approaches (DAs) to identifying potential skin sensitizers for regulatory purposes has been adopted by the Organisation for Economic Co-operation and Development (OECD) as Guideline 497 (OECD 2021a). A DA consists of a fixed data interpretation procedure that is used to integrate data from a defined set of information sources (OECD 2016). The data interpretation procedures provided in OECD Guideline 497 allow for a biologically relevant combination of multiple lines of evidence based on different key events in the adverse outcome pathway for skin sensitization. The DAs in Guideline 497 enable, without reliance on subjective expert judgment, both a determination of whether a substance should be considered a sensitizer or not (binary hazard classification) and a classification of a substance into specific sensitization potency categories per the United Nations Globally Harmonized System of Classification and Labelling of Chemicals (GHS) (UN 2021).

This manuscript represents the first in a series of three publications about an ongoing project to establish a database of HPPT results for use as human reference data to evaluate non-animal DAs for skin sensitization. Our specific aims were to (1) collect, curate, and evaluate published HPPT data; (2) document the reference chain for the data, which usually consists of several secondary and tertiary references; (3) annotate the data with structural information; and (4) make the data available to others in a user-friendly format for data mining, additional assessments of these data, model building, evaluating DAs, or evaluating other alternative test methods. A second publication will discuss the merits, limitations, and inherent uncertainties of using HPPT data to classify substances for skin sensitization hazard and potency according to the GHS (UN 2021) and propose modifications to the current GHS criteria to make better use of such data for chemical risk assessment. The final publication will characterize the variability and uncertainty in the HPPT data we have collected and evaluate the elements of the HPPT protocols and test reports that contribute to variability and uncertainty.

## Materials and methods

The starting point for our HPPT database was a data set compiled by the (U.S.) National Toxicology Program Interagency Center for the Evaluation of Alternative Toxicological Methods (NICEATM). The data set was originally compiled to support an evaluation by the (U.S.) Interagency Coordinating Committee on the Validation of Alternative Methods (ICCVAM) of the murine local lymph node assay for GHS categorization of substances for skin sensitization potency (ICCVAM 2011). The original NICEATM data set comprised 299 tests for 136 substances and was developed from references in the scientific literature and unpublished data submitted to ICCVAM by the Research Institute for Fragrance Materials (RIFM). Under the leadership of the German Federal Institute for Risk Assessment (BfR), this data set was greatly expanded, both in a number of records and in information captured, to support the OECD project on DAs for skin sensitization in Guideline 497 (OECD 2021a, b) and create the database described herein.

### Human predictive patch test types

The final HPPT database of 2277 tests includes two types of predictive patch tests: the HMT and the HRIPT. The database contains 1625 (71.4%) HMT and 652 (28.6%) HRIPT results. The designs for these tests are similar. In both, a test substance, dissolved or dispersed in a suitable vehicle of defined volume and concentration, is applied to a patch made of woven fabric. The patch is then applied to an extremity or back of a test subject and held in place by occlusive adhesive tape.^1^ The patch application is repeated a defined number of times during the induction phase, during which the body mounts an immune response if the test substance has skin-sensitizing properties. After a rest phase of 10–14 days, the test progresses to the challenge or elicitation phase during which a patch containing the test substance is again applied to the test subject. After a defined period of time, the test subject’s skin is observed for reactions indicative of an allergic response. Usually in the HMT, five induction exposures are applied to 25 test subjects over ten days. Conversely, in a typical HRIPT study, nine to ten induction exposures are applied to 50–200 test subjects over a period of three weeks. For nonirritating substances, the sensitivity of the HMT is increased by pretreatment of the exposure sites with sodium lauryl sulfate to enhance skin permeability and sensitivity of the test subjects by creating a state of mild irritation (Kligman 1966a). Because of concerns about both its potential to induce a severe skin reaction and its possible lack of scientific adequacy (Scientific Committee on Cosmetic Products and Non-food Products 2000), the HMT is no longer used.

We have distinguished three subtypes of the HMT and eight subtypes of the HRIPT (Table S1). The subtypes of the HMT vary in test volume, patch size, and site of induction. The subtypes of the HRIPT are more varied, differing in the number of subjects tested, test volume, patch size, duration of patch application, number of induction exposures, duration of the rest phase, number of challenge exposures, and duration of the challenge patch exposure.

### Evaluation of relative reliability

**Fig. 1:**
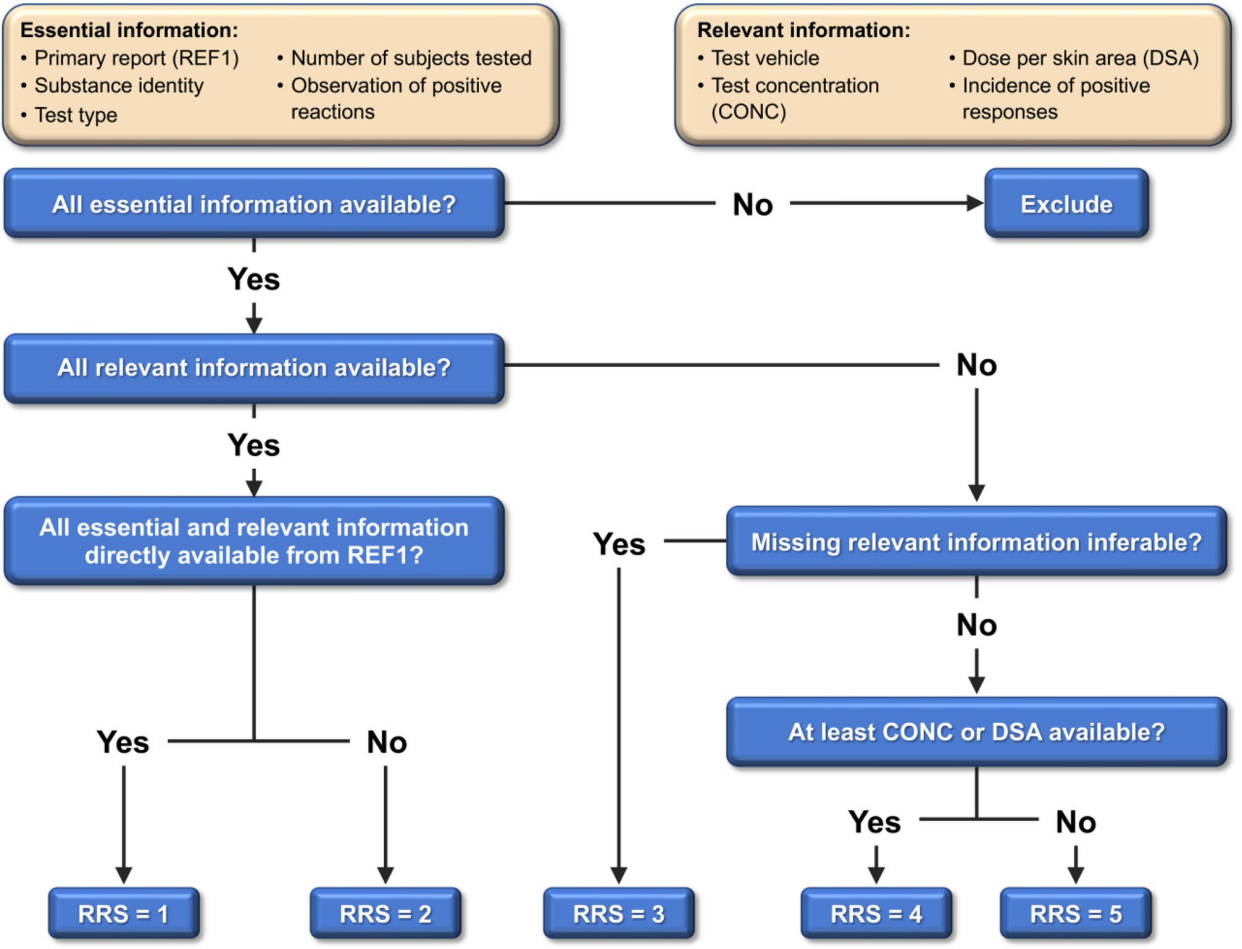
Relative reliability decision tree

The vast majority of the 2277 HPPT results in our database were generated decades ago using non-guideline, non-validated test protocols with no or unreported quality control. The primary reports were mostly unpublished, with raw data provided only to test sponsors such as RIFM. The test results in our database were therefore obtained from published summaries that usually did not provide complete protocol and results information; however, in some cases those can be obtained by inference. For example, the number of subjects sensitized can be calculated from % incidence. We used the information available to us to evaluate the quality of each test, focusing on the quality of reporting, rather than on the scientific quality of the experiment. We categorized the tests into five categories using a relative reliability score (RRS) according to the flow chart in Fig. 1. Lower scores correspond to higher reliability.

We first defined what we considered “essential information” necessary for a meaningful assessment. Essential information included availability or specification of (1) a primary report, (2) substance identity, (3) test type (HMT or HRIPT), (4) number of test subjects, and (5) information on whether positive reactions were observed. To meet the substance identity requirement, a Chemical Abstracts Service Registry Number (CASRN) or European Community Number (EC no.) would have to be specified. Some natural materials and extracts had multiple CASRNs/EC nos. specified without one single identifier being clearly assignable to the test material. In these cases, the substance was considered sufficiently described by the name of the source material (e.g., alantroot oil) or another commonly used name (e.g., Peru balsam). Each of the 2277 tests included in the final HPPT database was confirmed to have all essential information.

An RRS for each test having all essential information was then determined based on the degree to which it included further information relevant for the assessment. We defined “relevant” test information as the details about the dose and procedures applied during the induction process, including (1) the vehicle used, (2) the test concentration, (3) the dose per skin area (DSA), and (4) the incidence of positive responses. Although induction dose is typically reported as a percent (weight/volume) concentration, we calculated the DSA when it was not reported if adequate information (i.e., the applied volume of test material and the size of the test patch) was available to do so. DSA is a more appropriate dose metric than concentration because it aligns better with the incidence and severity of sensitization (Robinson et al. 2000; Kimber et al. 2008). Our selection of relevant parameters was guided by their availability in the references and is not exhaustive in terms of characterizing possible variability in test design. Other experimental parameters, e.g., the concentration used during the challenge/elicitation phase, may be very relevant for the test outcome but were in general not reported and therefore were not included in the determination of the RRS.

Following the decision tree illustrated in Fig. 1, the 2277 tests with essential information were assigned RRS as follows.

- RRS = 1 was deemed “highly reliable” because all essential and relevant information was available from the primary test report; 14.0% (318/2277) of the tests fell into this category.
- RRS = 2 was deemed “sufficiently reliable” because all essential and relevant information was provided either in the primary reference or in references cited by the primary reference (4.0% [91/2277] of tests).
- RRS = 3 was deemed “relatively reliable” because all essential but not all relevant information was available in either the primary reference or references cited by or citing the primary reference (71.6% [1630/2277] of tests). Missing relevant information was inferable from further available sources, e.g., from publications by authors with access to the primary reference, but with less confidence compared to tests with an RRS of 2.
- RRS = 4 was deemed “partly reliable” because all essential but not all relevant information was available in the primary reference or references cited by the primary reference (9.5% [216/2277] of tests). At a minimum, test concentration or DSA was available, but all missing relevant information was not inferable from additional sources.
- RRS = 5 was deemed “not reliable” because all essential information was provided but neither test concentration nor DSA were available (1.0% [22/2277]).

### Data fields

The goal of curating the HPPT data was to capture all the necessary information to characterize test design and quantitative test outcomes. Thus, the information captured by the data fields (Table S2) includes substance and structure identification, test type and subtype as specified in Table S1, test protocol details, test outcomes, RRS (as per Fig. 1), test remarks, and reference information. A quality control check of the test entries was performed by those who did not enter the data to ensure that entries matched the data in the associated reference.

For vehicle information, we noted the identity of the vehicle for each test. We obtained the values for vehicle density (Table S3) from chemical dossiers found in the European Chemicals Agency’s database of registered chemicals (ECHA 2022a). Vehicle density affects DSA calculation; an increase in vehicle density by 10% increases the DSA by the same relative amount. DSA values in the published literature are typically calculated assuming a density of 1 mg/μL, however, using the correct density for the vehicle provides a more accurate DSA value.

For all tests, we estimated the induction DSA if it was not provided by the source and if sufficient information was available. After dividing the percent concentration of the test chemical by 100 to convert to μg test substance/μg solution, we used the following equation to calculate the induction DSA:

$$ {\text{DSA }}\left( {\frac{{\mu {\text{g}}}}{{{\text{cm}}^{2} }}} \right) = \frac{{{\text{concentration }}\left( {\frac{{\mu {\text{g}}}}{{\mu {\text{g}}}}} \right)\,\, \times \,{\text{vehicle}}\,{\text{density}}\left( {\frac{{{\text{mg}}}}{{\mu {\text{L}}}}} \right) \times {\text{volume}}\,{\text{applied}}\,\,\left( {\mu {\text{L}}} \right)\, \times \,1000\,\left( {\frac{{\mu {\text{g}}}}{{{\text{mg}}}}} \right)}}{{{\text{patch}}\,{\text{size}}\,({\text{cm}}^{2} )}} $$

If DSA was provided by the source, we merely adjusted it for vehicle density. For positive tests, we also calculated the hypothetical induction DSA yielding one sensitized test subject (DSA1 +) by dividing the induction DSA by the number of sensitized subjects. Similarly, we estimated the hypothetical induction concentration (in percent) yielding one sensitized test subject (CONC1 +) by dividing the induction concentration by the number of sensitized subjects. The hypothetical induction DSA and concentration causing a 5% incidence of skin reactions (DSA05 and CONC05, respectively) were calculated by dividing the measured DSA or concentration by the measured incidence and multiplying the result by 5%.^2^ For example, a test with an induction DSA of 1400 µg/cm^2^ that produced an incidence of 2% positive reactions would provide an induction DSA05 of 3500 µg/cm^2^.

### Capture of reference citations

As a first step to documenting reference citations, bibliographic information and full texts of all RIFM monographs on fragrance ingredients as published in the peer-reviewed journal Food and Cosmetics Toxicology (January 1973–December 1981) and its successor Food and Chemical Toxicology (January 1982–December 2019) were gathered and stored in a reference library (EndNote™, v. X9.2). Next, this reference library was complemented with additional journal articles, book chapters, and reports from the published literature, which had been provided to BfR along with the original NICEATM database. Relevant publications noted by the authors within the references of the library or via other sources in the course of the project were also included in the library. A significant number of these references did not contain original test result data but cited original reports or other secondary sources. With every reference added, we checked whether the HPPT results cited therein were already part of the database, labeling each reference with its position in the citation chain (see the supplementary file for details). For example, the primary reference, which contained the original test data, was referred to as REF1. All other references were assigned positions in the reference chain (REF2, REF3, etc.) based on the sources cited. In addition to manual duplicate test result identification and data complementation accomplished by documenting the reference chain, we used in-house scripts generated with the statistical software “R” v.4.1.1 (R Core Team 2021). These scripts generated a duplicate-free list of references to streamline citation formats, producing a complete, chronological list of all references evaluated during the database compilation process that referred to each test result. This list can be used to track a test result cited in any of the publications back to its primary report.

### Availability of the HPPT database

The HPPT database is provided in two formats. It is available as a Microsoft Excel™ workbook on the NICEATM website at https://ntp.niehs.nih.gov/go/hppt. This version includes representations of each chemical structure in two formats: the Simplified Molecular Input Line Entry System (SMILES) and InChiKey, a condensed version of the International Chemical Identifier. The SMILES and InChiKey identifiers were mapped to the CASRN using data from the 10th incremental release of the U.S. Environmental Protection Agency’s CompTox Chemicals Dashboard at https://comptox.epa.gov/dashboard/ (Williams et al. 2017). The Excel workbook includes a worksheet containing the full list of all relevant references.

In addition to the Excel spreadsheet, the HPPT database is also available via NICEATM’s Integrated Chemical Environment (ICE) at https://ice.ntp.niehs.nih.gov/ (Abedini et al. 2021). Although the ICE format includes fewer data fields, it contains all of the relevant categorical and quantitative endpoints and this location is convenient for applying the analytical tools in ICE. The data fields in ICE include a single chemical name that is the preferred name from the CompTox Chemicals Dashboard, CASRN, test type, induction DSA, incidence (%) of positive responses, binary test outcome (active or inactive), DSA1 + (µg/cm^2^), DSA05 (µg/cm^2^), CONC1 + (%), CONC05 (%), and RRS.

### Chemical characterization

The variety of the substances included in the HPPT database was assessed using four different analyses. These analyses compared the substances in the HPPT database with the database of 196 chemicals used by the OECD to evaluate DAs for skin sensitization (“OECD chemicals”) (OECD 2021b) and with the universe of substances registered with the European Chemicals Agency (“EU chemicals”) (ECHA 2022b). The OECD chemicals represent a dataset of substances that have been frequently used to evaluate skin sensitization test methods, including new approaches. The EU chemicals represent the substances placed on the EU single market in quantities greater than 1 metric ton per year that were registered under Regulation (EC) 1907/2006 on the Registration, Evaluation, Authorisation and Restriction of Chemicals (REACH) as of April 5, 2022. Although 23,416 such substances were registered by that date, only 3,525 chemicals with CASRNs and SMILES could be used for this evaluation.

We compared the physicochemical properties of the chemicals in the HPPT database with those of the OECD and EU chemical sets. Predictions for molecular weight, vapor pressure, water solubility, LogP (log octanol:water partition coefficient), boiling point, and melting point were generated using the Open (Quantitative) Structure–activity/property Relationship App (OPERA v2.8) (Mansouri et al. 2018), which uses quantitative structure–activity relationship models to predict properties. The predicted physicochemical properties were evaluated using principal component analysis to compare the property coverage of the HPPT database compared with those of the OECD and EU chemical sets. The analysis was conducted using the prcomp function in R (v.4.0.2) and visualized using the autoplot function.

The structural variety of the HPPT database was also analyzed using ChemoTyper v1.0, a free software developed under contract with the U.S. Food and Drug Administration (Yang et al. 2015). ChemoTyper uses 729 chemotypes, which are generic structural fragments that represent chemical features, including connected and nonconnected chemical patterns as well as atom, bond, and molecular-based properties. We compared the frequency of each chemotype appearing in the HPPT database with those of the OECD and EU chemical sets.

A third analysis used the “Protein binding alerts for skin sensitization” profiler in QSAR Toolbox v.4.5 (OECD 2021c) to identify the mechanistic domains for the covalent interaction of each substance in the HPPT database with skin proteins. The profiler consists of structural alerts that are mechanistically justified or supported by experimental data (Yordanova et al. 2019). The results of this analysis were compared to those of a similar analysis of the OECD chemicals because both chemical sets have been used to evaluate test methods for skin sensitization. The frequency of appearance of each of the 11 protein binding domains in each dataset was displayed using the radar chart package in R software.

A fourth analysis of chemical variety compared the consumer use categories of the HPPT database to those of the EU chemicals using the Chemical Characterization tool in ICE v.3.6 (https://ice.ntp.niehs.nih.gov/Tools) (Abedini et al. 2021).

## Results

The initial HPPT database contained 2277 individual test results. Twenty-two of these had an RRS of 5 and were excluded from further analysis and characterization. Herein we characterize the available information for the remaining 2255 test results that had RRS of 1–4. Of those, 1619 were HMTs and 636 were HRIPTs. In the majority of the tests, no subjects were sensitized. Negative results were reported for 75.0% (1215) of the HMTs and 68.4% (435) of the HRIPTs. Twenty-five percent (404) of the HMTs and 31.6% (201) of the HRIPTs sensitized (i.e., returned a positive test result for) at least one subject. The most frequently used vehicle for both test designs was petrolatum (98.6% of the HMTs and 28.8% of the HRIPTs) (Table S4). The second most frequently used vehicle was diethyl phthalate (0.9%) for the HMTs and ethanol (13.4%) for the HRIPTs.

### Analysis of source references

In total, the database captures information from 2471 unique references, including 837 primary and 1634 non-primary reports. Only 33 (or 3.9%) of all primary reports were available to us, in contrast to 1522 (93.1%) of the non-primary reports.

**Fig. 2:**
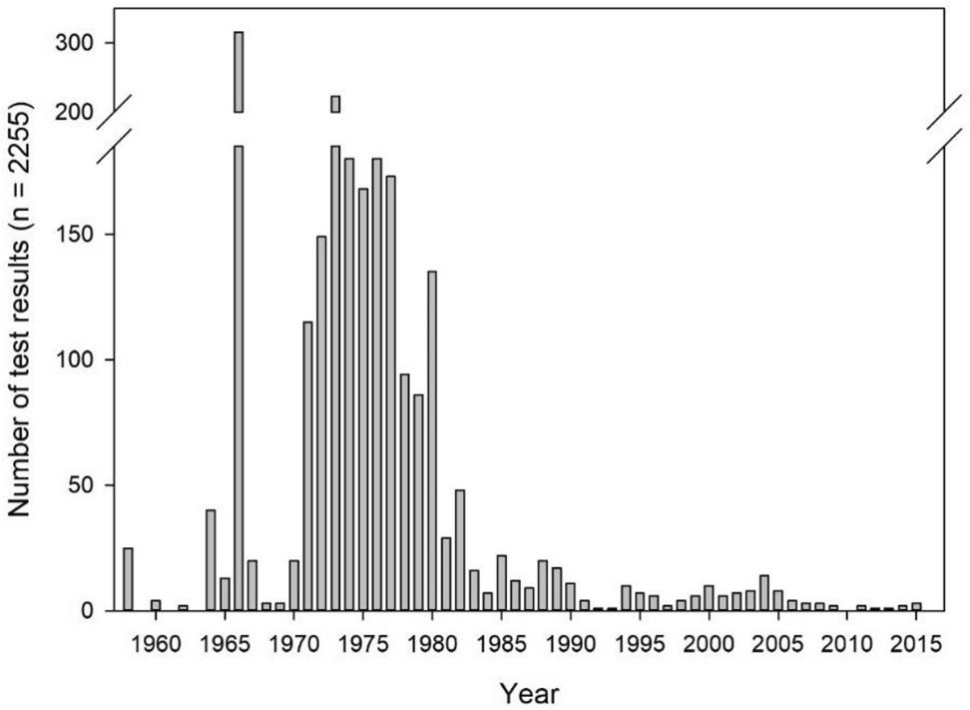
Distribution of primary reference dates for test results with RRS = 1–4 (*n* = 2255)

Figure 2 shows the distribution of publication dates over time for the primary references for all test results with an RRS of 1–4. These spanned a range of 57 years (1958 to 2015), with a large majority dating from the mid-1960s to the mid-1980s. Of the 2255 tests, 426 (18.9%) were conducted before 1970, 1814 (80.4%) before 1980, 2129 (94.4%) before 1990, and 2181 (96.7%) before 2000.

While we note above that only 3.9% of the primary reports were available to us, primary reports provided 535 (23.7%) of the 2255 test results with RRS of 1–4. This apparent discrepancy is explained by the fact that a number of primary references report multiple test results. For example, the three publications in which Kligman introduced the HMT in 1966 account for 314 of the 2255 test results with RRS = 1–4 (which also explains the peak for 1966 in Fig. 2) (Kligman 1966a, b, c).

As noted above, 1522 of the 1555 reports available to us were non-primary references. A considerable number of these publications contained multiple test results, often referenced by multiple parallel citation chains. This turned out to be a major hurdle for the data curation. The procedure we used to document the reference citation chains highlighted a number of shortcomings that could affect the overall quality of the reference data and, therefore, the reliability of the validation process as a whole (see specific examples in the supplementary file). Such quality issues are of particular concern in cases where later authors put their trust in these data as the foundation for method validation. Typical shortcomings include the following:

- No individual references are provided along with the reference values. For example, the data sources for DSA values are provided only via a blanket citation (e.g., "…an analysis of human data, adapted from references 23–43."
- Reference citations are inaccurate; references with no original data are cited or data in the cited reference does not match the values reported.
- Misspelled substance names, erroneous systematic names and/or inaccurate CASRNs are provided.
- Original values are processed further, e.g., by rounding, without providing transparent descriptions of the conventions used for the processing.

### Characterization of substances

**Fig. 3:**
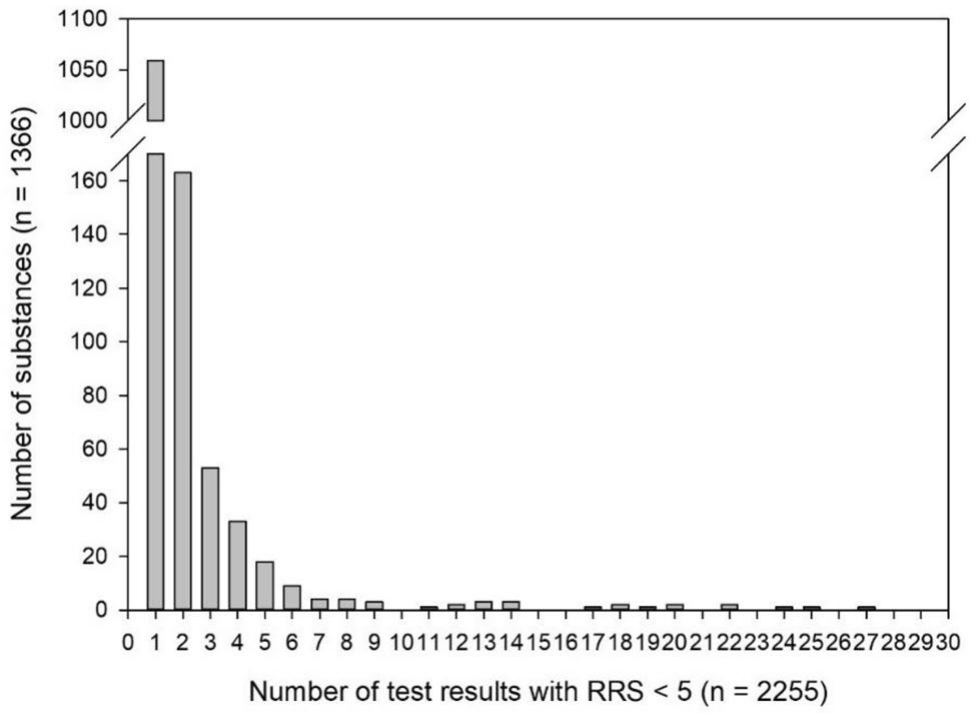
Distribution of the number of test results available per substance

The 2255 test results with RRS = 1–4 corresponded to 1366 unique test substances based either on CASRN or common name for complex substances such as “birch tar oil” or “parsley seed oil” that had no specific identifier available. The number of test results per substance ranges from 1 to 29 as shown in Fig. 3. For 1059 (77.5%) of the 1366 substances, only one test report was available. Two test reports were available for 163 substances, while 104 substances had between 3 and 5 test results. Between 6 and 29 test results were available for the remaining 41 substances.

Of the 1366 unique test substances with 2255 test results that were assigned RRS = 1–4, 1149 substances were associated with CASRNs. Those not associated with CASRNs were typically substances such as essential oils, natural product extracts, and mixed isomers. Of the 1149 substances with CASRNs, 1079 had associated SMILES structural representations.

**Fig. 4:**
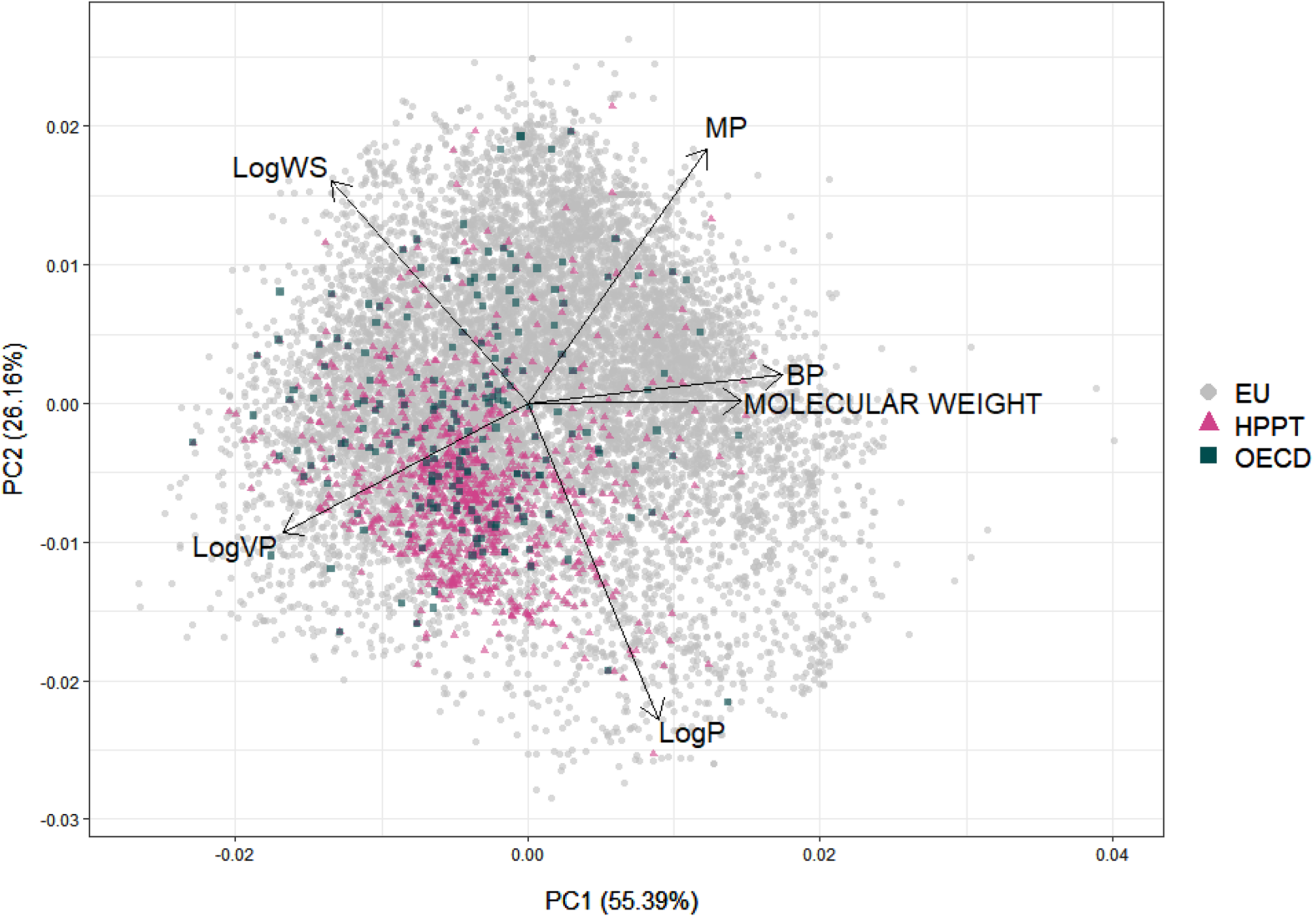
Principal component analysis plot of predicted physicochemical properties quantifying the coverage of the HPPT database and the OECD and EU chemical sets. *BP* boiling point, *LogP* log octanol:water partition coefficient, *MP* melting point, *WS* water solubility, *VP* vapor pressure

Figure 4 shows a principal component analysis biplot that compares the coverage of six physicochemical properties among the HPPT database and OECD and EU chemical sets. The HPPT database and OECD chemical set cover a similar space, with the much larger EU chemical set extending across all four quadrants of the plot. There is high overlap between all three chemical sets in the lower left quadrant, an area associated with higher vapor pressure and logP. Compared to the larger EU chemical set, chemicals that mapped to regions associated with higher water solubility, melting point, boiling point and molecular weight, are underrepresented in the HPPT database.

**Fig. 5:**
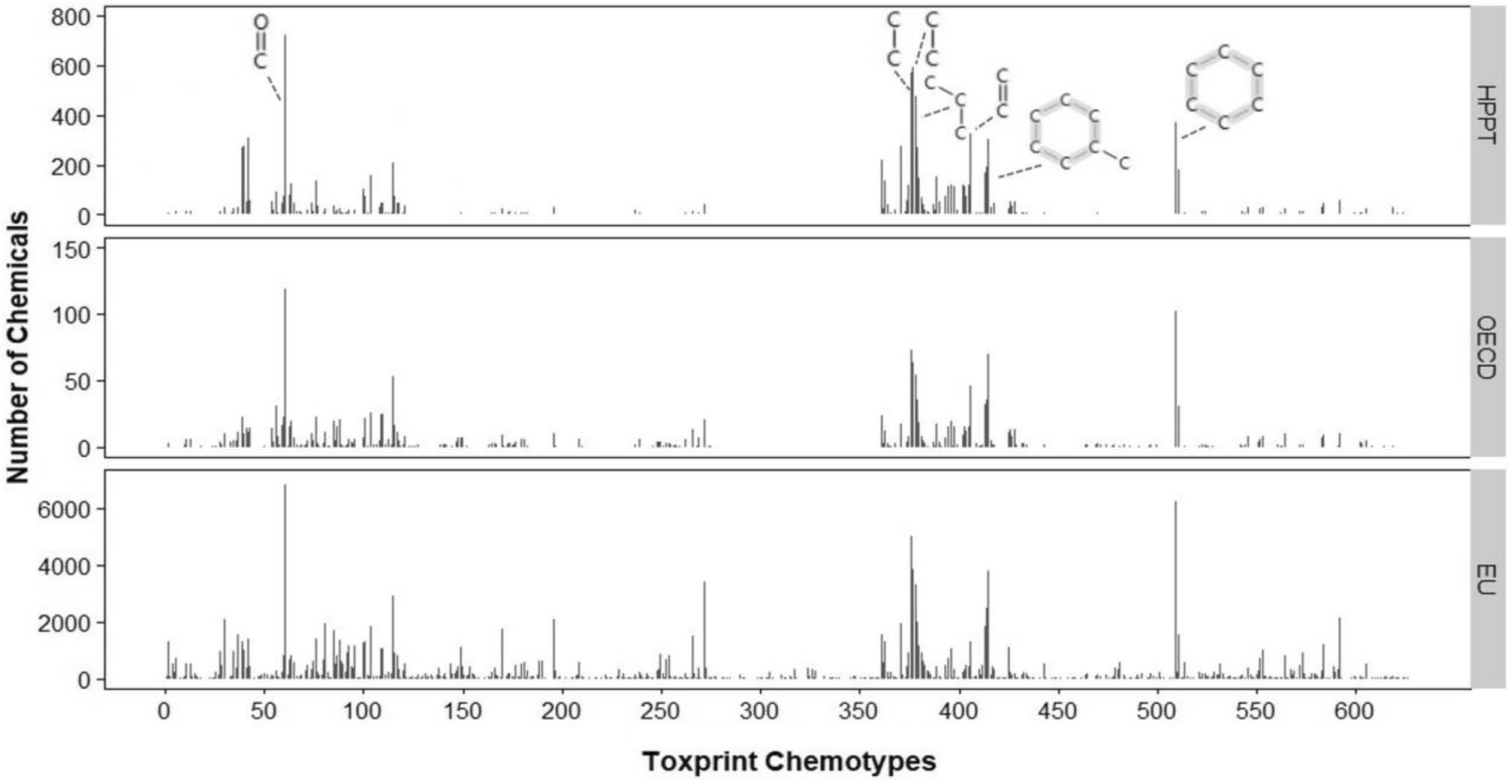
Frequency of 626 chemotypes in the HPPT database (top), OECD chemicals (middle), and EU chemicals (bottom)

Figure 5 shows the output of the Toxprint chemotype analysis by ChemoTyper (Yang et al. 2015), which compared the types of chemical structures in the HPPT database with those of the OECD and EU chemical sets by counting the frequency of chemical substructures. Chemotypes were available only for chemicals with associated SMILES: 1079 in the HPPT database, 196 in the OECD chemicals, and 13,525 in the EU chemicals. Of the 729 chemotypes available in Toxprint, 626 were represented in chemicals from at least one data set. These 626 chemotypes were given unique IDs, the remaining 103 chemotypes were removed from analysis.

The chemotype frequency profiles appear to be similar for all three datasets, with high frequencies for the following chemotypes (listed from left to right as appearing in Fig. 5):

- Bond:C=O_carbonyl_generic (chemotype 61).
- Chain:alkaneLinear_ethyl_C2(H_gt_1) (chemotype 376).
- Chain:alkaneLinear_ethyl_C2_(connect_noZ_CN = 4) (chemotype 377).
- Chain:alkaneLinear_propyl_C3 (chemotype 378).
- Chain.alkeneLinear_mono.ene_ethylene_generic (chemotype 406).
- Chain:aromaticAlkane_Ph-C1_acyclic_generic (chemotype 415).
- Ring.aromatic_benzene (chemotype 509).

However, the EU chemicals covered a broader chemical space than the HPPT database or the OECD chemicals; the EU chemicals included 625 chemotypes, the HPPT database included 317 chemotypes, and the OECD chemicals included 233 chemotypes.

**Fig. 6:**
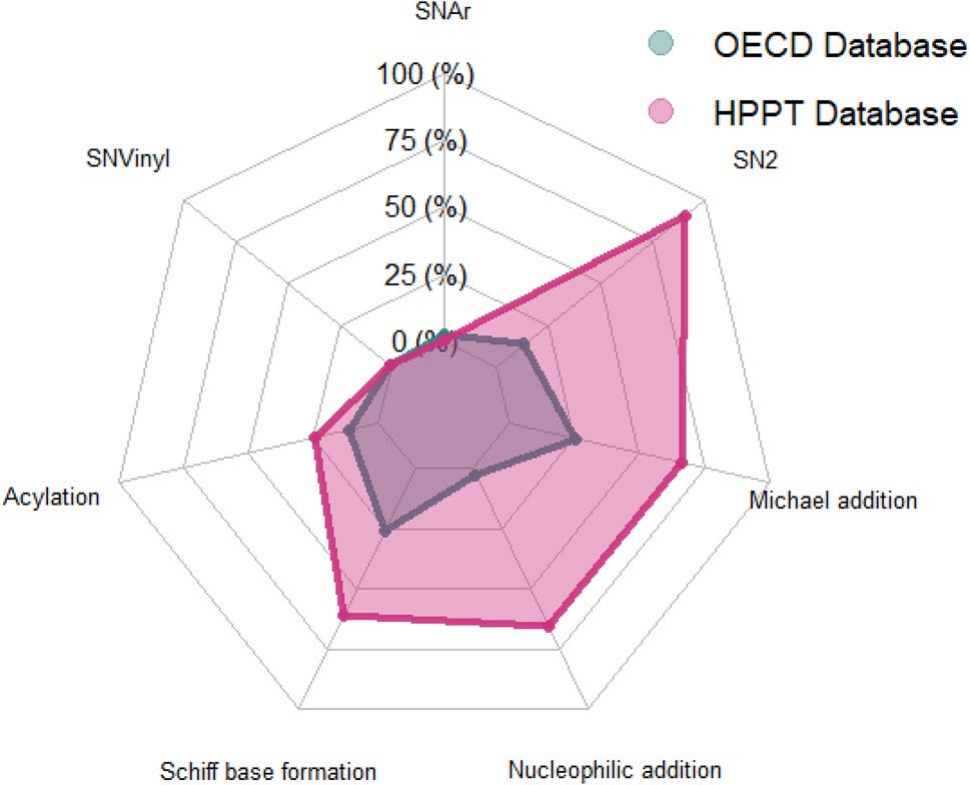
Analysis of protein binding alerts represented in the HPPT database (1079 substances) and OECD chemicals (196 substances) using the OASIS protein binding profiler provided by QSAR Toolbox v.4.5. The most frequent category represented in both datasets was “no alert” (716 for HPPT and 102 for OECD) and not shown in this figure

The mechanistic domains for the covalent interaction of substances in the HPPT database with skin proteins were characterized using the “Protein binding alerts for skin sensitization by OASIS” profiler in QSAR Toolbox v.4.5 (OECD 2021c) and compared with the OECD chemicals. The 1079 HPPT database substances with sufficient structural information and the OECD chemicals were categorized into seven of the 11 mechanistic protein binding domains (Fig. 6; binding domains not represented are not shown). A large majority of the substances in both data sets had no protein binding alerts (716/1079 for the HPPT database and 102/196 for the OECD chemicals). The most common domains for the HPPT dataset were SN2 nucleophilic substitution (109 substances), Michael addition (80), and nucleophilic addition (79) while the most common domains for the OECD dataset were Michael addition (31), Schiff base formation (31), and SN2 (16). Protein binding domains that were not represented included ionic interaction, radical reactions, SN1, and SN2 ionic.

**Fig. 7:**
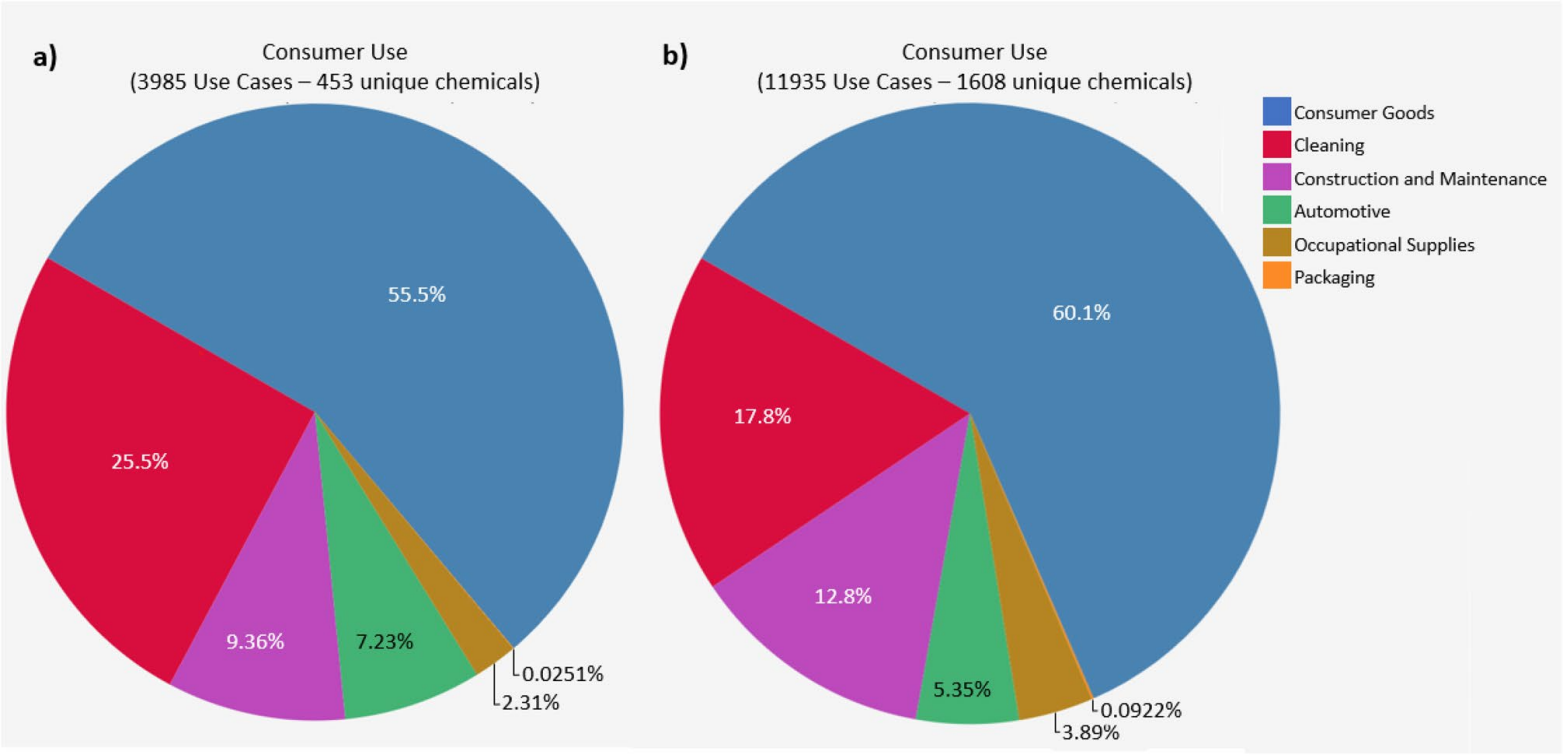
Consumer use categories as a percent of the total dataset for **a** 453 HPPT database chemicals and **b** 1068 EU chemicals

The Chemical Characterization tool in ICE v3.6 was used to characterize and compare product uses represented in the HPPT database and the EU chemicals. The tool had limited chemical coverage for both chemical sets: only 1608 of 13,525 EU chemicals (with CASRNs) were mapped to 11,935 use cases (unique chemical-use pairs), and 453 of the 1149 substances (with CASRNs) in the HPPT database were mapped to 3985 use cases. The consumer uses consist of six main sub-categories: automotive, cleaning, construction and maintenance, consumer goods, occupational supplies, and packaging material. For both chemical sets, most chemicals were mapped to the consumer goods sub-category, followed by cleaning products, construction and maintenance products, and automotive products (Fig. 7). Only a small percentage of EU and HPPT chemicals were associated with occupational supplies (less than 4%) or packaging (less than 0.1%). The proportion of chemicals in each of the consumer sub-categories was similar for the HPPT and EU chemical sets. The similarity of sub-category proportions may be due, however, to the over-representation of consumer-use products within the chemical characterization tool and limited chemical coverage rather than the true similarity between data sets.

## Discussion

The purpose of this data collection and evaluation effort was to provide human reference data for the evaluation of DAs for skin sensitization assessments for the development of OECD Guideline 497 (OECD 2021a, b). The resulting HPPT database has information organized in a standardized manner and enables tracking of each test’s reference chain, which allowed us to verify unique tests and correct for errors propagated by sequential citing over the years.

By mining RIFM monographs on fragrance ingredients and other literature available as of the end of 2019, we have collected data for 2277 individual tests. Of these, 2255 tests were deemed to have adequate test identification, dose, and effective reporting. The majority (72%; 1619/2255) of these tests were HMTs, only 27% (605/2255) of which produced at least one positive response. The preponderance of negative results may be due to the use of low doses in these tests, where the same chemicals might have displayed a sensitizing effect at higher concentrations. The use of low concentrations might possibly be explained by ethical concerns about inducing skin sensitization in humans who have no known allergy to a substance or by the fact that often the safety of in-use concentrations (plus a safety margin of e.g., tenfold) was assessed rather than trying to establish sensitization potential in absolute terms. HRIPTs performed for RIFM in recent decades were performed only to confirm a non-sensitizing dose (Politano and Api 2008). However, this may not be the case for earlier HRIPTs or for HMTs, and thus there could be other explanations for the large number of negative tests, including that they could simply reflect the prevalence of non-sensitizing chemicals in general.

Our characterization of the substances in the HPPT database showed that the included substances represent a range of physicochemical properties (Fig. 4) and structural features (Fig. 5). The variety of physicochemical properties was similar to but not quite as broad as, that of the EU chemicals, which are intended to represent marketed substances from a diversity of product sectors. Our evaluation of Toxprint chemotypes indicated that while the EU chemical set contained a number of structural features that were not represented in the HPPT dataset, the HPPT, OECD, and EU datasets were remarkably similar with respect to chemotypes appearing at high frequency. Likewise, an analysis of protein binding alerts by QSAR Toolbox showed similarities between the HPPT dataset and the OECD chemicals, while an evaluation of consumer use data by the ICE Chemical Characterization tool showed similarities between the HPPT database and the EU chemicals.

In recent years, the so-called reproducibility or replicability crisis has been subject to considerable debate in the scientific community (Nelson et al. 2021). While this discussion is typically in the context of new experimental findings that cannot be reproduced by other or even the original authors (Baker 2016), it also applies to the validation of new toxicological test methods. The extent to which new methods reproduce reference results obtained with previous “gold standard” methods cannot be appropriately evaluated unless the reproducibility of the reference data have been characterized. There are a number of sources of variability for HPPTs, such as variability in nominally identical test substances, variability within and between test populations (e.g., population size, age, skin characteristics), variability in induction and challenge exposure protocol, and variability in scoring the test results (OECD 2021b). In a future manuscript, we will thoroughly characterize the variability of these data and the sources of variability with respect to the test information collected (i.e., test type, vehicle, physicochemical properties, etc.). We will also demonstrate and discuss the generally high reproducibility of HPPT-based GHS classifications, specifically with respect to distinguishing sensitizers from chemicals that do not require classification for skin sensitization under the GHS.

Moreover, all validation efforts should start with rigorous quality control of the underlying reference data, for which accurate and comprehensive reporting of the test results in question is indispensable. The first and foremost prerequisite for accurate and comprehensive reporting of test results is the traceability of the results to their original sources. During our curation of a comprehensive set of HPPT data from the published literature, we encountered numerous obstacles, including highly complex multi-link and parallel citation networks, that make quality control of the reference data a highly tedious and time-consuming endeavor. As a result of our experience with this extensive data curation process, we recommend the following quality standards:

- When authors reference test results from other publications, the original/primary report should always be identified and referenced; indirect citations should be avoided.
- Authors should avoid publishing work based on data for which the original reports or other reliable reports by the original authors are not available to them. In cases where original reports were not available, this should be made transparent.
- Each result cited from a publication should be individually linked to its source. Reference statements such as "test results 1–10 were taken from references A-E" should be avoided.
- Experimental parameters with an impact on the variability or reliability of the test results should be reported as extensively as possible (supplementary files will suffice).
- Where reference data from other publications are used, a quality assessment system should be implemented and transparently documented.

Adherence by authors to these standards and their implementation by academic journals as quality criteria in their peer review process would significantly facilitate the use of their data by other scientists, particularly when these data are used as reference data for method validation purposes.

## Summary

We have compiled a database of 2277 HPPTs that provides protocol elements, reliability scores, positive or negative outcomes, sensitization rates, and calculated traditional and non-traditional dose metrics for each test data point. Chemicals are cross-referenced with multiple names and identifiers, making chemicals of interest easy to find. Chemical structural information such as SMILES and InChiKeys are provided for modeling purposes. The database is publicly available on the NICEATM project page at https://ntp.niehs.nih.gov/go/hppt to serve as a resource for additional evaluation of alternative skin sensitization methods and the development of new approach methodologies for skin sensitization assessments. The data are also available on NICEATM’s ICE website, allowing interested users to explore them using the ICE suite of data analysis tools.

In addition to the present article, we plan to publish two additional manuscripts based on these HPPT data. One manuscript will discuss the regulatory use of these data for classifying substances for skin sensitization hazard and potency according to the GHS (UN 2021). This manuscript will recommend modifications to the current GHS classification criteria and provide a workflow for classification along with an interactive web implementation using R software. Another manuscript will leverage over 200 substances in the database that have multiple test results to evaluate the variability of these data. This manuscript will review and discuss the elements of these protocols and test reports that contribute to variability and uncertainty.

## Supplementary Information

Below is the link to the electronic supplementary material.

Supplementary file1 (DOCX 229 KB)

## Abbreviations

BfR: German Federal Institute for Risk Assessment
CASRN: Chemical Abstracts Service Registry Number
CONC1+: Hypothetical induction concentration yielding one sensitized test subject
CONC05: Hypothetical induction concentration yielding 5% incidence of skin reactions
DA(s): Defined approach(es) to testing and assessment
DSA: Dose per skin area
DSA1+: Hypothetical induction DSA yielding one sensitized test subject
DSA05: Hypothetical induction DSA yielding 5% incidence of skin reactions
EU: European Union
GHS: United Nations globally Harmonized System of Classification and Labelling of Chemicals
HMT: Human maximization test
HPPT: Human predictive patch test
HRIPT: Human repeat insult patch test
ICCVAM: Interagency Coordinating Committee on the Validation Of Alternative Methods
ICE: Integrated Chemical Environment
LogP: Log octanol:water partition coefficient
NICEATM: (U.S.) National Toxicology Program Interagency Center for the Evaluation of Alternative Toxicological Methods
NIEHS: National Institute of environmental health sciences
NTP: (U.S.) National Toxicology Program
OECD: Organisation for Economic Co-operation and Development
REACH: Regulation (EC) 1907/2006 on the Registration, Evaluation, Authorisation and Restriction of Chemicals
RIFM: Research Institute for Fragrance Materials
RRS: Relative reliability score
SMILES: Simplified Molecular Input Line Entry System

## Footnotes

1 The HPPT database also includes tests that used Hill Top Chambers®, which use a different system of occlusion compared with occluded patches, but the patch materials are similar.

2 The concept of DSA1 + /CONC1 + and DSA05/CONC05 and its use in classification under the GHS will be explained and discussed in more detail in the second publication of this series.
