# Supplementary material for: A database of human predictive patch test data for skin sensitization
Source: Arch Toxicol. 2023 Aug 24;97(11):2825–37. doi: 10.1007/s00204-023-03530-3 (PMC10504114; doi:10.1007/s00204-023-03530-3)
Supplement: Supplementary file 1 — Supplementary file1 (DOCX 229 KB) [file 204_2023_3530_MOESM1_ESM.docx]

Table S1. Overview of test method subtypes in the HPPT database

| **Test type^a^** | **Sub-type^a^** | **Typical number of test subjects** | **Induction** | | | | | | **Typical rest phase (d)** | **Challenge** | | **Reference** |
| --- | --- | --- | --- | --- | --- | --- | --- | --- | --- | --- | --- | --- |
|  |  |  | **SLS^b^ pre-treatment** | **Test volume (µL)** | **Patch size (cm^2^)** | **Contact per exposure (h)** | **No. of exposures** | **Site of induction** |  | **No. of exposures** | **Contact time per exposure** |  |
| HMT (1619^c^) | Klig66  (178) | 25 | yes | 1000 | 14.5 | 48 | 5 | forearm or lower leg | 10^d^ | 1 | 48 | (Kligman, 1966) |
|  | Klig6675  (528) |  |  | 1000 or 300^e^ | 14.5 or 4.0 ^e^ |  |  | forearm, back, or lower leg |  |  |  | (Kligman, 1966; Kligman and Epstein, 1975) |
|  | Klig75  (785) |  |  | 300 | 4 |  |  | forearm or back |  |  |  |  |
| HRIPT (636^c^) | Shel53  (11) | 200^f^ | no | not reported | not reported | 24 | 10-15 | not reported | 14-21 |  | 48 | (Shelanski and Shelanski, 1953) |
|  | Voss58  (24) | 50-60 |  | 500 | 4.2 |  | 9 | upper arm | 10 |  | 24 | (Voss, 1958) |
|  | Drai59  (73) | 200 |  | 500 | 6.5^g^ |  | 10 | arms or back | 10-14 |  | 24 | (Draize, 1959) |
|  | Gri69  (3) | 60-70 |  |  | 3.9 |  | 9 | upper arm | 17 |  |  | (Griffith, 1969) |
|  | MM73  (91) | 200 |  |  | 6.5^h^ | 48-72 | 10 | arm | 14 |  | 72 | (Marzulli and Maibach, 1973) |
|  | JK77  (10) | 150 |  | 500 | 4 |  | 9 | upper back |  | 2 | 48 | (Jordan Jr. and King, 1977) |
|  | MM80  (45) | 200 |  | 200 | 2.54 |  | 10 | arm or upper back |  | 1-2 | 72 | (Marzulli and Maibach, 1980) |
|  | RIFM08  (67) | ≥ 100 |  | 300 |  | 24 | 9 | back | 10-14 |  | 24 | (Politano and Api, 2008) |

^a^ Parentheses show the number of test results with Relative Reliability Score less than or equal to 4. Of the 2277 tests, 2255 met this criterion. Only 22 tests had Relative Reliability Score of 5. (Higher reliability scores indicate lower reliability.)

^b^ SLS: sodium lauryl sulfate, an irritant used for pre-treatment in the HMT to maximize sensitization outcome.

^c^ The discrepancy between this value and the sum of the subtypes is explained by the presence of test results for which the test design could not be assigned (i.e., the test type is provided as “other” in the database).

^d^ According to Greif (1967).

^e^ We used the average ratio of test volume/patch size of 72 µL/cm^2^ for calculations herein. The original HMT (Kli66) was published in Kligman (1966c). However, Kligman and Epstein (1975) modified that design, reducing the patch size from 14.5 to 4 cm^2^ and the test volume from 1 to 0.3 mL (Kli75). Thus, there is uncertainty about the degree to which the original design had been modified for tests performed between 1967 and 1974 (Kli6675). Using the average of Kli66 (69 µg/cm^2^) and Kli75 (75 µg/cm^2^) introduces a small error of 4% vs. a potential greater error of approximately 9% when erroneously assigning Kli66 or Kli75.

^f^ Not reported in original publication, but in Kligman (1966) and Marzulli and Maibach (1976).

^g^ The value used in calculations reported here is 6.4516 cm^2^, i.e., one square inch.

^h^ Not reported in the original papers, but inferred after communications with the Research Institute for Fragrance Materials and Dr. Maibach.

**Table References**

Kligman AM (1966): https://doi.org/10.1038/jid.1966.160

Kligman AM, Epstein W (1975): https://doi.org/10.1111/j.1600-0536.1975.tb05389.x

Shelanski HA, Shelanski MV (1953): A new technique of human patch tests. Proceedings of the Scientific Section of the Toilet Goods Association 19, 46-49.

Draize JH (1959): https://babel.hathitrust.org/cgi/pt?id=uc1.b3596550;view=1up;seq=5

Griffith JF (1969): https://doi.org/10.1016/S0041-008X(69)80014-1

Marzulli FN, Maibach HI (1973): https://library.scconline.org/v024n07/

Marzulli FN, Maibach HI (1976): https://doi.org/10.1111/j.1600-0536.1976.tb02972.x

Marzulli FN, Maibach HI (1980): https://pubmed.ncbi.nlm.nih.gov/7441083/

Jordan WP, King SE (1977): https://doi.org/10.1111/j.1600-0536.1977.tb03582.x

Politano VT, Api AM (2008): https://doi.org/10.1016/j.yrtph.2007.11.004

Voss JG (1958): https://doi.org/10.1038/jid.1958.120

Table S2: Database structure

| **Section** | **Fields included** |
| --- | --- |
| Substance identification | Chemical name and synonyms, Chemical Abstracts Service Registry Number, European Community number; DSSTox Substance Identifier, from the Distributed Structure-Searchable Toxicity (DSSTox) Database (Williams et al. 2107 https://comptox.epa.gov/dashboard) |
| Structure identifiers | Simplified Molecular-input line-entry system (SMILES) code; InChi key, a condensed version of InChi string, which is the IUPAC International Chemical Identifier that represents chemical structure |
| Test type | HPPT or HMT and subtype from **Table S1** |
| Test protocol details | Volume of test material applied, area of skin patch, vehicle, density of vehicle, number of test subjects, concentration of test item applied, and induction dose per skin area |
| Test outcomes | Number of test subjects with a positive response, incidence of positive response as a percentage, call (binary test outcome of active or inactive), estimated induction concentration producing one sensitized test subject, estimated concentration causing an incidence of 5%, and estimated induction dose per skin area causing an incidence of 5% |
| Reliability | Relative reliability score (RRS |
| Additional test information | Test remarks (for example, the location of test information in the cited reference, the purity of the test substance, pooled results, and inaccuracies) and reference information |

Table S3: Densities for vehicles used in the HPPT database

| **Vehicle** | **Value(s) provided in ECHA^a^ database (mg/µL)** | **Value used in HPPT database (mg/µL)** |
| --- | --- | --- |
| Acetone | 0.79 at 20 °C | 0.8 |
| Diethyl phthalate (DEP) | 1.118 at 20 °C | 1.1 |
| Dimethyl phthalate (DMP) | 1.194 at 20 °C | 1.2 |
| Dimethyl sulfoxide (DMSO) | 1.1 at 20 °C | 1.1 |
| Ethanol (EtOH) | 0.78933 at 20 °C | 0.8 |
| Polyethylene glycol (PEG) | 1.116 at 20 °C | 1.1 |
| Petrolatum | 0.77 – 0.96 at 15 °C | 0.9 |
| Triacetin | 1.161 at 20 °C | 1.2 |
| Water | - | 1.0 |
| Others (density unknown) | - | 1.0 |

^a^https://echa.europa.eu as of 2019-06-22

For mixtures of the above, individual densities were weighted according to their relative proportion, e.g. DEP/EtOH 3:1 = (3 x 1.1 + 0.8)/4 = 1.0. Nevertheless, it must be acknowledged that these values are estimates and the true density of the test items used in HPPT testing is not known (also considering the possible impact of dissolving the test item in the vehicle), adding further uncertainty.

**Table S4: Distribution of vehicles used for tests in the HPPT database**

| **HMT (1619 tests)** | **Number of tests** | **% HMTs** |
| --- | --- | --- |
| Diethyl phthalate | 15 | 0.93% |
| Ethanol | 2 | 0.12% |
| Hydrophilic petrolatum | 1 | 0.06% |
| Petrolatum | 1596 | 98.58% |
| Not reported | 1 | 0.06% |
| None | 1 | 0.06% |
| Petrolatum + sodium dodecyl sulfate | 2 | 0.12% |
| Water | 1 | 0.06% |
|  |  |  |
| **HRIPT (636 tests)** | **Number of tests** | **% HRIPTs** |
| 1% Aqueous Tween 20 | 1 | 0.16% |
| 1% Sulfur in polyethylene glycol | 1 | 0.16% |
| Petrolatum and then water ^a^ | 1 | 0.16% |
| 65% Ethanol | 1 | 0.16% |
| 95% Ethanol | 5 | 0.79% |
| 99% Petrolatum + 0.5% diethyl phthalate | 1 | 0.16% |
| 99% Petrolatum + 0.5% diethylene glycol monoethyl ether | 1 | 0.16% |
| Acetone | 9 | 1.42% |
| Aqueous ethanol | 2 | 0.31% |
| Corn oil | 1 | 0.16% |
| Cream | 1 | 0.16% |
| Cream base | 2 | 0.31% |
| Diethyl phthalate | 26 | 4.09% |
| Diethyl phthalate: ethanol 3:1 | 38 | 5.97% |
| Diethyl phthalate: ethanol 3:1 + alpha tocopherol | 3 | 0.47% |
| Diethyl phthalate: ethanol 3:1 + triethyl citrate(97:3) | 1 | 0.16% |
| Dimethyl phthalate | 25 | 3.93% |
| Dimethyl sulfoxide | 2 | 0.31% |
| Ethanol | 85 | 13.36% |
| Ethanol: diethyl phthalate | 1 | 0.16% |
| Ethanol: diethyl phthalate 3:1 | 41 | 6.45% |
| Ethanol: diethyl phthalate 3:1 + alpha tocopherol | 1 | 0.16% |
| Mineral oil | 2 | 0.31% |
| None | 1 | 0.16% |
| Not reported | 93 | 14.62% |
| Perchloroethylene | 1 | 0.16% |
| Petrolatum | 183 | 28.77% |
| Petrolatum + triacetin | 1 | 0.16% |
| Petrolatum or 5% aqueous Tween 85 | 2 | 0.31% |
| Specially denatured alcohol 39C | 70 | 11.01% |
| Specially denatured alcohol 39C: diethyl phthalate 3:1 | 6 | 0.94% |
| Specially denatured alcohol 40 | 3 | 0.47% |
| Triethylcitrate: diethyl phthalate:ethanol 3:1 | 1 | 0.16% |
| Vaseline | 1 | 0.16% |
| Water | 23 | 3.62% |

^a^ Six induction exposures used petrolatum as the vehicle and four used water

^b^ The report is unclear on which vehicle was used for this particular test.

The HRIPT used the widest variety of vehicles, 35, while the HMT used eight different vehicles. The most frequently used vehicle for both tests was petrolatum (98.6% of the HMTs and 28.8% of the HRIPTs). The second most frequently used vehicle for the HMT was diethyl phthalate (0.9%). The second most frequently used vehicle for the HRIPT was ethanol (13.4%), but the vehicle was not reported for 14.6% of the HRIPTs.

**Reference Checking Procedure for the HPPT Database**

With every reference added to the database, we checked whether the HPPT results provided therein were already part of the database. To assist with the identification of duplicate tests, we used the following procedure to document the reference chain:

- If a reference contained original test data, it was termed the “primary reference,” or “REF1”.
- If it did not contain original data, we examined whether REF1 was cited as the source for the data, in which case the secondary reference was identified as REF2. References not citing REF1, but only REF2 were identified as REF3, and so on.
- In each case, the whole citation chain was documented by adding all references linking the one under examination with REF1. This allowed us to identify and remove duplicate test results from the database. Sometimes, we could also obtain complementary information about the test design or outcome from later references in cases where, e.g., REF1 was not publicly available or previous references had incompletely reported these data, but the information was accessible to the authors of the later publication.
- Primary references (REF1) were noted in a specific database field. A separate field was added to document whether REF1 was publicly available.
- Information on secondary references (REF2) was stored in an additional database field. These references were tagged as “#1#”, “#2#”, etc., in chronological order, with the first author’s name used as a secondary sorting criterion for references with the same year of publication.
- Similarly, tertiary reports (REF3) citing those secondary references were captured in chronological order and tagged accordingly. For example, the chronologically second REF3 citing the REF2 tagged as #5# for a given test result was tagged as “#5#2#.” This procedure was continued for higher-level references in an analogous way, up to the level of REF9, which was the longest reference chain observed.

This scheme is illustrated by the example of an HMT result for nickel sulfate. The original data were published in reference no. [27] (the identity of this publication is not relevant here), which was identified as REF1. This original report was directly cited by 10 further references in the database (REF2 level), which were therefore tagged as #1# to #10#. The secondary reference [873], tagged as #5#, was cited by three further citations at the REF3 level, which were therefore tagged #5#1# to #5#3#, and so on (Table S5). Establishing these citation chains allowed us to correct for transmission errors along citation chains, typographic errors, etc., and to complement originally incomplete test result data with information from later publications.

Table S5 Scheme used to annotate citation chains

| **REF1** | **REF2** | **REF3** | **REF4** | **REF5** | **REF6** |
| --- | --- | --- | --- | --- | --- |
| [27] | #1# [103] |  | | | |
|  | #2# [910] |  |  |  |  |
|  | #3# [869] |  |  |  |  |
|  | #4# [219] |  |  |  |  |
|  | #5# [873] | #5#1# [1066] | #5#1#1# **[874]** | #5#1#1#1# **[2224]** |  |
|  |  |  | #5#1#2# **[875]** | #5#1#2#1# **[874]** | #5#1#2#1#1# **[2224]** |
|  |  | #5#2# **[874]** |  | | |
|  |  | #5#3# **[875]** |  |  |  |
|  | #6# **[874]** |  | | | |
|  | #7# **[875]** |  |  |  |  |
|  | #8# [876] |  |  |  |  |
|  | #9# [872] |  |  |  |  |
|  | #10# [877] |  |  |  |  |

Illustrative example of a test result for nickel sulfate described in the text. For simplicity, this example shows only tertiary and lower-order references citing REF2 #5#, with citation chains of the other secondary references not shown. Bold text shows references cited by both references in multiple levels of the citation chains.

Soon after starting this work, it became obvious that references often cited the same test result multiple times, either directly by citing the original primary test report (REF1) or indirectly by citing other secondary (tertiary, etc.) references in parallel. In the example in Table S5, this is the case for references [874], [875], and [2224]. In this way, some of the references were mapped to the same test result by multiple citation chains. For example, four citation chains connect reference [874] to reference [27], the primary reference (REF1) for test result no. 1770 (not shown in Table S5). We used the reference chain documentation to visualize the complexity of the citation networks using Cytoscape v.3.9.0 (Shannon et al. 2003)

As noted in the text of the manuscript, 1522 of the 1555 reports available to us were non-primary references. A considerable number of publications contained multiple test results, often referenced by multiple parallel citation chains. This was a major hurdle for the data curation. We present here an example that demonstrates some of the problems we encountered in the process and may serve to raise awareness of how specific shortcomings affect the overall quality of the reference data and therefore the reliability of the validation process as a whole. Similar shortcomings were found in many references; therefore, we have anonymized the authors’ names here and simply call the publication “Review 1.” Review 1 was cited by evaluations that were instrumental in preparing the OECD DAs evaluation (OECD 2021).

Review 1 classified a set of chemicals according to their sensitization potential on the basis of human reference data. Review 1 provided no-observed-effect levels (NOELs), i.e., the maximum known DSA values at which no sensitization in exposed humans was observed, for 56 substances classified as sensitizers or nonsensitizers. A further 24 substances were classified in this review as nonsensitizers, without NOEL values, while for another 51 substances, the data were designated as not sufficient for the development of NOELs. Capturing data from this publication and aligning it with our database turned out to be highly resource-intensive for the following reasons:

- The nature of the tests (HMT, HRIPT, or other human data, e.g., diagnostic patch test data) behind the NOEL values was not reported.
- For each NOEL reported, one key reference is cited; however, some of these references were primary reports, while others were review publications that contained no original data. Moreover, in some cases, references were cited in error, in that the cited references did not contain data matching the reported NOEL values or, sometimes, any data at all for the specific substance.
- The data sources used for classifying substances as nonsensitizers were only provided via a blanket citation (e.g., "...an analysis of human data, adapted from references 23-43"). Notably, some of these references also contained data on the substances for which NOELs were cited. In another case, the outcome “not sensitizing” was not traceable to any of the references cited.
- In a number of cases, the key references did not contain information about the NOEL or the negative outcome reported. In some cases, the NOELs could not be deduced from any other reference in the reference list. In particular, for twelve of the NOEL values, the key reference was a short conference abstract containing no human data at all. For dimethyl fumarate, the authors provided a NOEL of 88 µg/cm^2^ that was not found in the key or any other reference from the reference list. In fact, we were not able to identify any HPPT result for this substance. We found a value only for its congener, diethyl fumarate.
- In one case, the substance identification was not accurate: the data for nickel were actually obtained with nickel sulfate with the DSA converted to the nickel mass fraction. Moreover, the reported NOEL of 154 µg/cm^2^ was actually identified as a *lowest*-observed-effect level (LOEL) in the key reference.
- In other cases, such as for cobalt sulfate, the authors of Review 1 considered the available human test data insufficient to define a NOEL in spite of the fact that HPPT data were available from publications documented in the reference list.

A second anonymized reference, herein referred to as “Review 2,” provided a supplementary data file that contained human reference data from only two sources: Review 1 and a previous publication from the Review 2 authors’ own group, “Article 3” (Fig. S1a). Of note, neither Review 1 nor Article 3 contained original HPPT data. As Fig. S1b shows, only Review 1 directly cites a number of primary references, while Article 3 does not.

| 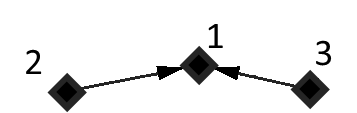 | 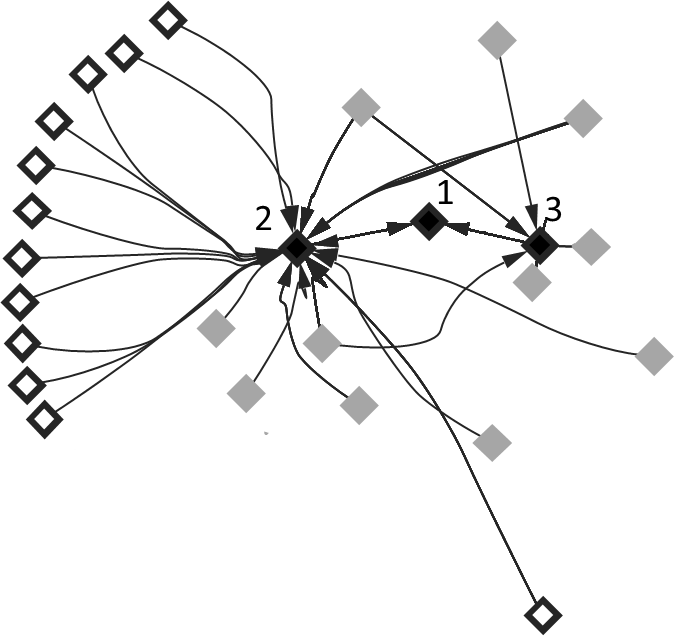 | 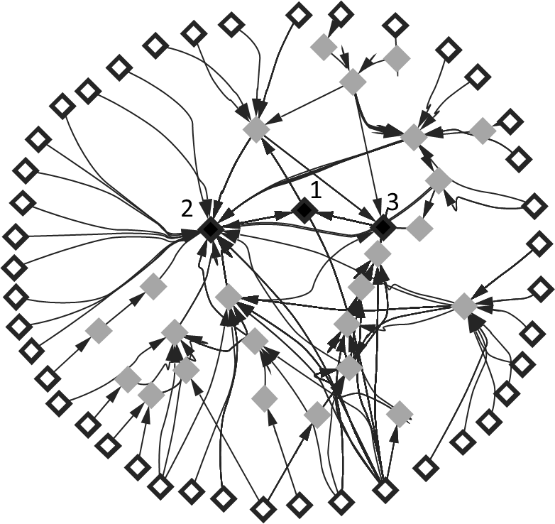 |
| --- | --- | --- |
| (a) | (b) | (c) |

Fig. S1. Origin of the human reference data cited in the supplementary data file of Review 2. a) The supplementary data file for Review 2 (node 1) cites only two references, Review 1 (node 2), and Article 3 (node 3), all represented as filled black diamonds. Note that one edge symbolizes two connected references. b) Looking at the references cited directly by two of the publications, Review 1 cites 12 primary references (diamonds with white interior and black border) and eight non-primary references (gray diamonds), while Article 3 cites only five non-primary references and no primary references. c) The full citation network reveals 42 primary references with only 12 directly cited by Review 2; some are connected to the citing publication of Review 2 by a citation chain as long as six references.

The highly complex citation network representing the source of the human reference data described in Review 2 involving multi-link and parallel citation chains is depicted in Fig. S1c.

From these figures, a number of conclusions can be drawn:

- With the exception of a few test results that are directly mentioned in the main text of Review 2, the authors seem to have based their performance assessment on reference data for which the primary reports were not available to them.
- Consequently, they had no choice but to rely on the authors they cited to have performed an adequate and thorough quality assessment of the underlying data.
- A number of publications have discussed relevant aspects of HPPT quality, including the early Kligman (1966 a,b,c) papers and a handful of other original papers, such as Kligman and Epstein (1975) or Marzulli and Maibach (1980). However, such discussions are mostly absent from the papers that form the citation network of Review 2, as are clear descriptions of the quality assessment work performed and the criteria for inclusion and use in that context.
- Finally, these factors make it unclear how a meaningful peer review of these publications (i.e., Reviews 1 and 2 or Article 3) was possible, given that the reference data were not even available to the authors themselves.

Such quality issues are of particular concern in cases where later authors put their trust in these data as the foundation for method validation. We noted similar deficiencies in a significant number of other publications, even some cited as key references in Review 1. Typical shortcomings are, for example:

- No individual references are provided along with the reference values.
- Misspelled substance names, erroneous systematic names and/or inaccurate CASRNs are provided.
- Original values are processed further, e.g., by rounding, without providing transparent descriptions of the conventions used for the processing.

**References**

Kligman AM (1966a): https://doi.org/10.1038/jid.1966.158

Kligman AM (1966b): https://doi.org/10.1038/jid.1966.159

Kligman AM (1966c): https://doi.org/10.1038/jid.1966.160

Kligman AM, Epstein W (1975): https://doi.org/10.1111/j.1600-0536.1975.tb05389.x

Marzulli FN, Maibach HI (1980): https://pubmed.ncbi.nlm.nih.gov/7441083/

OECD (2021) https://www.oecd-ilibrary.org/environment/guideline-no-497-defined-approaches-on-skin-sensitisation_b92879a4-en

Shannon P, Markiel A, Ozier O, et al. (2003) https://doi.org/10.1101/gr.1239303
